# 6.E. Skills building seminar: ‘Think global, act local'- using the Place Standard tool to support action on climate and health

**DOI:** 10.1093/eurpub/ckac129.358

**Published:** 2022-10-25

**Authors:** Organised by: EUPHA-HIA, EUPHA-ENV, Chair persons: Odile Mekel (EUPHA-HIA), Peter Van Den Hazel (EUPHA-ENV)

## Abstract

The global determinants of health act on our physical, social, and economic environments or “places”, which impact on population health, wellbeing and inequalities. Climate change poses an increasing threat to health and equity. So tackling the consequences of the climate emergency is one of the most serious issues facing our places and it will lead to a transformation of people's lives. Good place-making is essential for designing a robust response to the climate emergency at a local level, such as taking local action to cut emissions (mitigation) and to increase resilience to local climate change impacts (adaptation). A place-based approach to addressing climate has the potential to deliver co-benefits to drive fair and just solutions that also support health, wellbeing, and equality. The Scottish Place Standard Tool (PST) is an effective and widely used tool originally created in a collaborative partnership between Public Health Scotland, Scottish Government, Architecture & Design Scotland, and Glasgow City Council for assessing a place, with a focus on health and wellbeing. As it syntheses complex evidence into 14 easily understood questions to develop a shared understanding of what matters in a place, it has proved to be an effective tool to facilitate a place-based approach, in which local communities, local government and third sector can work together to deliver improvements. Today it is used widely in Scotland, its application internationally has extended across 14 countries in Europe and worldwide it has been translated into at least 16 different languages. Whilst climate has been strengthened in the core content of the tool, both Scotland and Germany have recognised the need of additional support to really focus on climate issues and have embarked on separate simultaneous projects to enhance PST's ability to do so. The workshop aims to introduce the PST with a climate lens and share the different approaches from Scotland and Germany.

The objectives of the workshop are to:

• Provide a brief overview of the connection between climate, place, health and health equity.

• Introduce and compare recent work in Scotland and Germany, which both aim to build on the success of the original PST to strengthen climate considerations in the design and delivery of healthy and equitable places.

• Enable participants, through sharing learning from real-life examples gathered during piloting of the new PST climate resources, to consider how they might practically engage with climate and health through a place-based approach.

This will be achieved through an introduction to the key principles behind the PST, and presentations from Scotland and Germany on their respective approaches to strengthening the potential of the PST to address climate, health and equity, plus practical case study examples of climate action supported by the PST. Time will be provided at the end for practical involvement of the participants and for discussion.

**Key messages:**

• The climate emergency is one of the biggest challenges facing public health. The Place Standard Tool addresses the need to include climate change and health issues within a discussion about a place.

• Adding a “climate lens” to the Place Standard Tool offers the potential to maximise co-benefits to drive fair & just solutions that support health, wellbeing, & equality.

